# Elite darts performance and the social influence of real crowds and simulated crowd noise

**DOI:** 10.1038/s41598-023-39378-x

**Published:** 2023-07-31

**Authors:** Jona Greve, Edda van Meurs, Bernd Strauss

**Affiliations:** Department of Sport and Exercise Psychology, Institute of Sport and Exercise Sciences, University of Muenster, Horstmarer Landweg 62b, 48149 Muenster, Germany

**Keywords:** Human behaviour, Psychology

## Abstract

While the effect of missing audiences has been studied numerously in team sports with diverse effects, studies on individual performances are rare. The current investigation analyzes performances of professional dart players in (a) the absence of spectators, (b) the presence of real crowds, and (c) artificial crowd noise (simulated crowds) substituting live spectators during the COVID-19 pandemic. Empirical evidence suggests that performances in coordination-based accuracy tasks are negatively impacted by the presence of others. Therefore, we hypothesize that performance of elite darts players deteriorates in the presence of a real audience (RA) in comparison to no audience (NA) and simulated audience (SA). https://dartsorakel.com provided the data of professional tournaments played from 2018 to 2021, which included *N* = 26,724 individual performances from *k* = 442 players (98.8% male). How RA and SA impacted checkout percentage (CP) and three-dart average (3DA) was analyzed using separate multilevel models, adding various control variables. Competing with audiences (SA and RA) resulted in decreased CP with an effect of β_stand_real_ = − .20, *p* < .001, and β_stand_sim_ = − .14, *p* < .001. 3DA increased with SA, β_stand_sim_ = .08, *p* < .001, and decreased with RA, β_stand_sim_ = − .07, *p* < .001. The results show that real crowds appear to have a negative impact on darts performance, yet effect sizes are small.

## Introduction

At the end of 2022, during the annual PDC (Professional Darts Corporation) world darts championship tournament at the sold-out, world-famous Alexandra Palace in London (Ally Pally), something remarkable happened: during the quarter-final match between Welsh player Gerwyn Price (world-rank No 1) and German player Gabriel Clemens (world-rank No 25), Gerwyn Price appeared to be distracted by the shouting and whistling of the crowd. Price, who is generally used to playing in front of huge crowds that are not in his favor^1^, was unable to focus on the match and proceeded to wear over-ear, and later in-ear, hearing protection trying to block out the noise of the audience^2^. While engagements with the audience in darts are common, this instance is unprecedented. Price lost the match regardless of this measure. The disruptive behavior of the crowd seemed to have a negative impact on his performance and distracted him to a point where he was unable to focus on throwing accurately, a motor task that needs concentration and coordination^3^.

Ironically, in the 2021 world darts championship, the same German player, Gabriel Clemens, won against the 2020 world champion Peter Wright in the third round. However, that game was played without any live spectators but with a simulated audience, where players would hear a crowd via speakers, yet no spectators were present, as 2 years ago, the PDC darts world championship had to be played mostly without a live audience because of the worldwide COVID-19 pandemic. Peter Wright, as he said himself, was not able to “switch on” during the game, as it “felt like [he] was playing at home”^4^.

The investigation of the positive but also negative social influences of active and passive sport spectators or observers on performances of motor tasks is one of the oldest topics in sport psychology, sport sciences and sport economics (for a recent overview see e.g., the chapter by Strauss et al.^5^ or Wallace et al.^6^).

With the COVID-19 pandemic starting in spring 2020, audiences were widely excluded from most of the sport events worldwide. A prominent example was the postponement of the Olympic games 2020^7^, which were nonetheless held without spectators^8^. This often called unique “natural experiment”^9,10^ has led to many recent studies, mostly investigating the home field advantage and the impact of crowds on team outcome and refereeing decisions^11,12^ in team sports such as soccer^10,13^, and American sports such as baseball^14^, basketball^9,15^, football^16^, or hockey^17^. Overall, there is a large variation in the results of these studies, and the direct impact of the absence of spectators on match outcomes and team performances are inconclusive (e.g., with respect to an increase, decrease or no-change of the home advantage), whereas it seems that an absent audience can reduced the referee bias^10^. However, the effect of absent audiences on individual performances in team sports is rarely investigated. The limited evidence suggests that players’ individual performances within a team do not change significantly, as has been shown in rugby^18^. In soccer, the absence of crowds had a positive or negative effect on individual penalty kick performances, depending on whether the team was performing at home or away, respectively^19^. Outside of team sports, such an analysis on the impact of the absence of spectators due to the COVID-19 pandemic on individual-sports performances has not yet been conducted.

This archival study presents data and results regarding performances over a span of 4 years (2018–2021) in the individual sport of darts, which can be considered a coordination-based task^20^, requiring high levels of accuracy^21,22^. The main focus of the current manuscript is to study the social influence of a large audience on dart throwing in elite darts players. Darts allows to compare performances of elite players as they compete without an audience present and also in the presence of a larger crowd, as professional tournaments are played under both conditions. Moreover, due to the unique approach by the PDC, regarding the COVID-19 pandemic, to replace the real audience with pre-recorded artificial crowd noise (simulated audience), this study allows to investigate the effects of such a condition, in which the audience is not present, but the players (as well as the TV viewers) hear the sound of a simulated audience played loudly through large speakers in the venue, yet nobody is actually spectating. The simulated audience even adapted their singing and the volume as a reaction to the performances of the players.

### Darts

Darts is played by throwing three small arrows, usually made of tungsten, into a dartboard that is divided into 20 segments each worth a different number of points. Each segment additionally has a double field and a treble field (doubling and trebling the points of the segment they belong to). The ’oche’, the distance from where the players throw on the board, has a length of 237 cm and the board hangs on a wall at a height of 173 cm. Professional tournaments are played in the *501-up* format in which players begin with 501 points and have to reach exactly zero points by hitting a double field with the last dart^23,24^. If achieved, this is called a *checkout* and the player wins one *leg* (comparable to a *game* in tennis). A darts match is usually played over a fixed distance (e.g., best of 11 legs). An in-depth explanation of the rules of darts can be found in previous reports^23,25,26^, and the exact rules are described in the darts regulation authority rulebook^24^. During matches, the rules are enforced by a *“Caller”*, which is responsible for the correctness of the scores and calling the scores to the opponent. The caller also has the function of a referee, in case players violate the rules of the game. Some professional darts competitions under the umbrella of the PDC are either held in the absence of spectators, meaning that the players are competing alone in small cubicles, with only a caller present. Other tournaments are played on a stage, usually in the presence of a larger crowd (apart from the time during the COVID-19 pandemic, where the crowds were replaced by simulated crowd noise). The social condition under which the players compete is dependent on the type of tournament.

### Theoretical background

Research in the field of social facilitation (i.e., performances when others are merely present) found that simple or well-learned tasks tend to be facilitated, while performance in difficult or novel tasks decreases^27–29^. Additionally, the presence of an audience tends to increase performances in motor tasks that place demands on the condition of performers, but seems to decrease some performances that require precise object manipulation^30,31^. These findings were recently confirmed in a systematic review and meta-analysis of 100 years of research on social facilitation during motor tasks^32^. Several different theories have been developed to explain the results either based on the drive/activation level of the performer^27,28,33,34^, or based on the attentional conflict that arises when others are present^35–37^.

Studies that include supportive or non-supportive audiences (i.e., when an audience is not only merely present but actively behaving, e.g., jeering, cheering, booing, etc.) instead of passive spectators are expected to display a similar trend of results^6^. Condition-based (effort-based) performances often increased with external verbal encouragement^38^. On the other hand, coordination-based (skill based) sports performances were found to be negatively affected by the behavior of the audience^39^, however, in an experimental study^40^ results of cheering, jeering and silence on various coordination-based sports tasks (i.e., basketball free throws, golf pitches and baseball pitches) were inconclusive: golfers were strongly negatively affected by jeering and cheering, whereas the results in the basketball task were similar in all audience conditions.

Apart from the positive effect of crowd support in the form of encouragement for condition-based tasks, in research pertaining to “choking under pressure”, supportive crowds can be a source of social pressure, which could increase the tendency to “choke” in some athletes, thus resulting in decreased performance^6^. “Choking under pressure” is described as “inferior performance despite individual striving and situational demands for superior performance” (p. 610)^41^. Baumeister^41^ argued that the importance to perform well leads athletes to attempt to ensure correct execution by monitoring the performance process. This paradoxically reduces performance, since monitoring well-learned tasks—which do not require conscious monitoring—interferes with the automated processes developed through practice. Choking under pressure has been shown in various coordination-based tasks^42^, and interventions to combat the negative effects of choking have been developed^43–45^.

There are a few studies which investigated choking under pressure in darts^23,25^. Interestingly, different studies found that professional darts players overall do not display the tendency to choke in important situations: when investigating the likelihood of winning one “leg” (i.e., fixed distance within a game that wins a point) of darts when the opponent exerts pressure on the performer (by threatening to win the leg with the next throw), players did not display decrements of performance^23^ and even increased their performance^25^. However, another study^41^ did find a “home choke”, i.e., that playing in a venue close to their home town (< 100 km) significantly impacted darts players’ performance: they experienced significant decrements in performance when having the advantage of throwing first in a darts match, yet the effect did not carry over merely competing in their home country (i.e., further away from their home town)^46^. Ötting et al.^23^ have applied social-facilitation theories to explain these results and argued that pressure is perceived differently, i.e., that the pressure exerted from a (supportive) crowd differs from the within-match variation to perform well or when competing for monetary rewards. Players experience a “home choke”^46,^ but their performance does not decrease when the opponent generates pressure within a leg of darts. This supports the idea that dart players experience different forms of pressure. Ötting et al.^23^ therefore suggest comparing darts performance in situations where the players perform in front of an audience and without spectators present.

Some professional dart tournaments on the PDC circuit are played in front of large and noisy audiences and are broadcasted on TV. The best example is the already mentioned PDC world championship in London at Alexandra Palace with several thousand live spectators. Other tournaments are held without any spectators in a silent environment (except for a referee calling the scores).

Meanwhile, the audience in darts broadcasts is a major source of entertainment for television viewers. Therefore, the PDC broadcasted tournaments with artificial crowd noise during the COVID-19 pandemic, i.e., the players and television viewers could hear crowd noise, yet no one was present at the venue^47,48^. Artificial crowd noise has been utilized in various sports such as rugby or football, but for the TV viewers only^49^, but no studies to date have examined a possible effect of the artificial cheers and jeers that are played back to the performers. Darts offers the unique opportunity to study audience effects in three ways: without an audience, as most tournaments of the year are played behind closed doors unavailable to spectators, with a real audience, players competing on stage with people watching the performance, and with artificial crowd noise, only hearing a crowd, whilst no spectators are actually present. These tournaments are held regularly and were also held before the COVID-19 pandemic. Additionally, darts offers several benefits in investigating individual responses^46^. Darts performance is objectively measurable and the task is highly standardized with little variation. The minimal influence of the caller prevents subjective referee decisions, thus eliminating a referee bias, which is often found in social-influence literature^10^. Clear rules regulate the height of the board, and even apply restrictions of dart weight and length. Additionally, the large amounts of data that are available allow us to correct for inter-individual differences between the players^23^.

### Research question

The present investigation aims to answer the question of how elite darts players perform (1) *in the absence of crowds* (i.e., the absence of spectators or noises) and in the presence of (2) *real crowds* (i.e., the presence of active spectators) to study the social influence of a large, active crowd on a coordination-based task with accuracy requirements. This is unique, because the influence of a real audience on darts performance has not been studied to date. With the changes undertaken by the PDC (i.e., replacing a live audience with simulated crowd noise during the COVID-19 pandemic), an additional research question arises, which is the impact of (3) *simulated crowds* (i.e., artificial crowd noise as a substitution of real crowds in the venue). The impact of simulated crowds is a novum in research on social influence of spectators on motor tasks. The current research answers pending questions within the field of darts and other sports, where the impact of crowds is vividly discussed^1,50^. Based on the results from research in the field of social influence and previous research in darts, we hypothesize that performance would be the lowest when a real audience is present due to the disruptive potential of active spectators while performing a coordination-based sports tasks that requires accuracy and concentration.

## Methods

### Data

The current archival investigation analyzes data over 4 years of professional darts tournaments that took place under the umbrella of the PDC (https://www.pdc.tv). The PDC organizes the most important professional tournaments (e.g., World Darts Championships in London, the UK Open, the Pro Tour, the European Tour etc.), mostly located in the UK but also worldwide. Typically, a PDC tournament consists of several qualified players who are competing against each other in subsequent knock-out rounds in a single-elimination format.

Data was made available for the purposes of this current study by the business company *Darts Orakel*, UK (https://dartsorakel.com/), the current partner of the UK television company Sky television. Their professional and protected website contains information on players, such as a current form ranking, as well as detailed stats of previous performances and tournaments. The data was retrieved using RStudio version 1.4.1106 with the R package rvest^51^. The data has been scraped directly from the website, which is used commercially. No manual data extraction took place, thus reducing the amount of user error. Random data points have been checked, by comparing the individual data points to the results displayed on television and other websites. Data of all the ranking tournaments played from January 2018 to March 2021 were used, including the year, tournament name, player, tournament stage, as well as the performance variables CP and 3DA for each match.

#### Three-dart average

The three-dart average (3DA) is the most commonly used and most accurate performance indicator in darts^26,46^, and was also used for the current investigation. 3DA is the average number of points that are scored per throwing visit. Requiring 15 darts to finish a leg of 501 points results in a 3DA of 100.2 points on average with each turn. In a newsletter issue from June 2021, the creators of the results-publishing website *Darts Orakel* have reported a correlation of *r* = .85 of 3DA and matches won over a period of 4 years^52^, indicating the reliability and validity of 3DA as a performance measure.

#### Checkout percentage

The other commonly used performance indicator in darts is checkout percentage (CP)^25,26^. CP describes a player’s percentage of successful double field hits to finish a leg. If a player scores a double on their first try every time, their CP is 100%. CP can be considered to have a higher content validity in comparison to 3DA but should be as reliable due to the smaller number of throws and attempts in a leg.

#### Player strength

Additionally, to control for the player’s strength, two covariates were retrieved by the website darts1.de^53^: the Pro Tour Order of Merit (1-year ranking points earned in Pro Tour and European Tour events exclusively) and the Order of Merit rank (2-year world rank based on the money earned in all ranking tournaments) at the end of each year. Including all tournaments in the Order of Merit results in large gaps between the rankings, as some tournaments pay out larger price pools (e.g., the prize money for winning the World Darts Championship is £500,000, whereas the winner of a Pro Tour event gets £12,000). The Pro Tour Order of Merit is considered to be a more accurate measure of current level of play by some professionals, as it is less influenced by these large outliers^54^.

#### Audience conditions

Per tournament, it was logged manually whether it was played with no audience, a real audience, or with a simulated audience (i.e., with artificial crowd noise). Most tournaments are played behind closed doors, unavailable for spectators. These Pro Tour events are only played with a referee present and are not televised (a livestream for a paid subscription is available). Other tournaments in darts are played with audiences that typically host 3000 to 5000 spectators who are booing, cheering, wearing costumes, singing, etc. In rare occasions, up to 20,000 spectators are present^55^. With the surge of the COVID-19 pandemic in 2020, spectators were no longer allowed at the venues for sporting events. To maintain a pleasant viewer-experience, the PDC included artificial crowd noise (i.e., players and television viewers could hear an audience that was pre-recorded and played via speakers at the venues). Artificial audience reactions could be matched to the situation in the game (e.g., cheering when somebody throws a 180). Thus, apart from the referee and two neutral markers writing down the scores, the players were competing alone.

The data availability from 2018 to 2021 was highest for competitions when the players played with *no audience* (*n* = 21,032), followed by the condition with a *real audience* (*n* = 4894) and lowest for the *simulated audience* condition (*n* = 798, only for 2020 and 2021 during the pandemic). These numbers are individual performances, i.e., performance of one player in a match-up. Therefore, the *no audience* condition was chosen as a baseline, as data availability was highest.

#### Round

Players in later stages are presumed to have higher performances than in earlier stages of the tournament, as players who play well progress further in the tournaments. Thus, we included the round of the tournament in the current analysis as a performance predictor. Round indicates the number of players who are still actively competing in the tournament.

### Statistical analysis

Multilevel modeling, or hierarchical linear modeling, was used to account for performances nested within the players. Multilevel models are extended forms of regression approaches to account for grouping structures or clusters within the data that are not present on a single level^56,57^. We used multilevel modelling as we are interested in individual performances (level 1) nested within 1 year (level 2) nested within the same individual (level 3). This allows us to account for the fact that individual performances and performances within the years are likely to be correlated, and therefore to control for individual differences in multiple performances. For the current investigation, hierarchical linear models were used to model the performance parameters CP and 3DA separately as level 1-outcome variables. Level 1 and level 2 predictor variables (performance predictors) were added with each subsequent model. The data was analyzed using the *lme4* package^58^ in R.

#### Modeling checkout performance

A hierarchical linear model was used to model the data using CP as the level 1-outcome variable to investigate the impact of audience type on checkout performance. The “random intercept model” for checkout performance including individual players as random effects was used to test whether significant clustering was present. The year of performance was added as a level 2-variable, to account for nesting within the year of performance nested within the player. The type of audience condition was the main predictor of interest. Since it is a categorical predictor (no audience, simulated audience, real audience), the “no audience condition” was chosen as a baseline due to the high availability of data and the impact of simulated and real audience was added in subsequent models, which then gradually included the following control variables: the ranking of the players (in both the “Order of Merit” and the “Pro Tour Order of Merit”) to control for skill level, and the “round of the tournament” (i.e., last 128 players, last 64 players, etc.), as players in later stages of a tournament are presumed to perform better than in earlier stages. Model fit parameters (i.e., Log-Likelihood) were compared and significant control variables were retained in further models building the final model. Partially standardized beta coefficients as effect sizes were obtained according to Lorah^59^.

#### Modeling three-dart average

**Figure 1:**
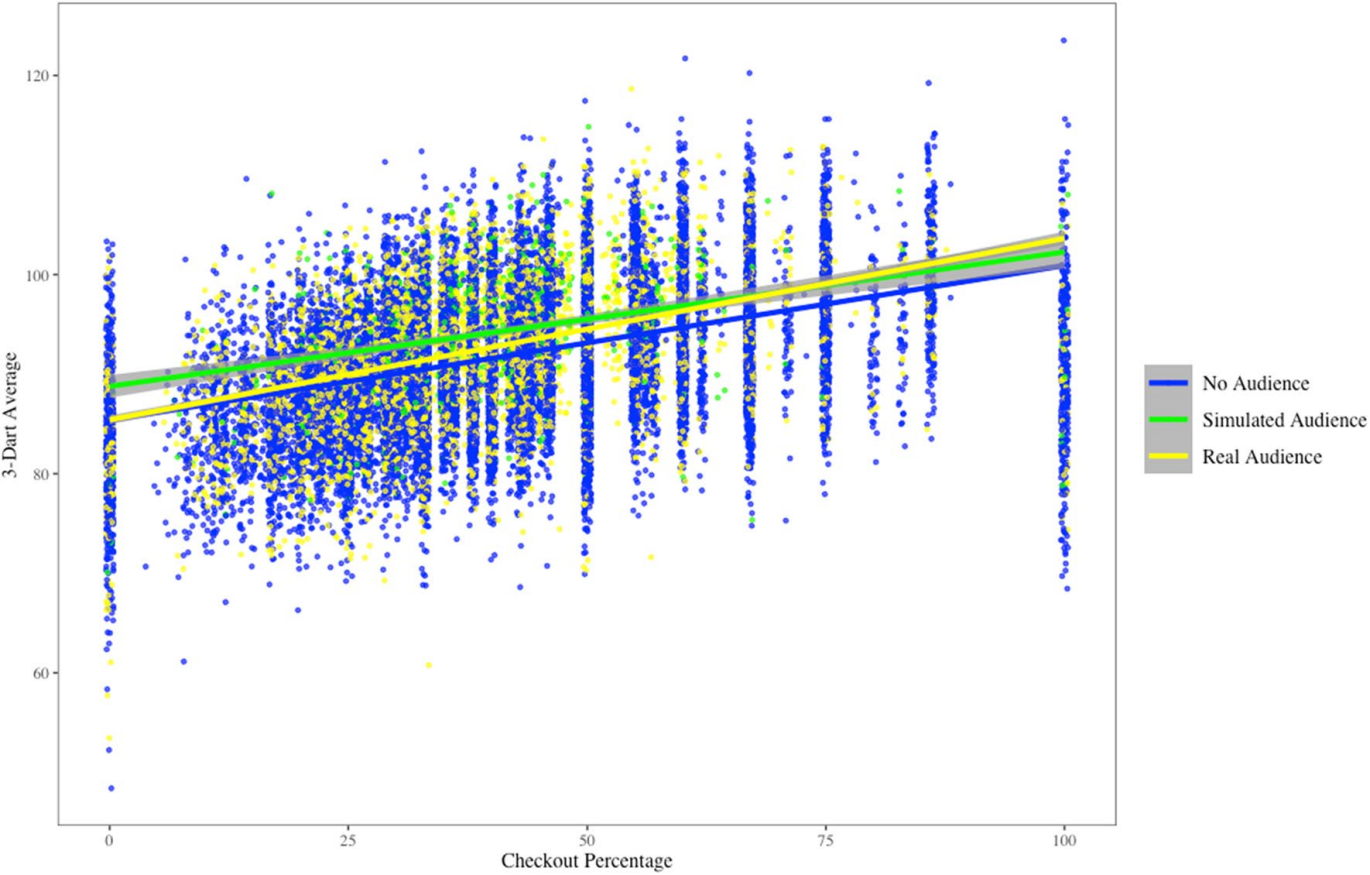
Linear relationship between 3DA and CP for the three audience conditions.

Modeling 3DA followed the same procedure as the model for CP. In addition to the predictors that were added for the first hierarchical linear model, CP was added as a level 1-predictor, since CP and 3DA correlate moderately, *r* = .41, *p* <  .001. Controlling for the variance of CP in 3DA allows for more accurate inferences independent of CP. A visual representation of the relationship between CP and 3DA can be found in Fig. 1.

#### Supplementary analyses

Additional analyses using various subsets of the available data with more strict exclusion criteria were performed to function as robustness checks of the main model. Two analyses only included the top 32 Pro Tour Order of Merit players and top 64 Order of Merit players. Those were carried out to eliminate a possible bias in the data through elite players with very high ranks. Better players are usually playing in tournaments with real audiences and simulated audiences (as replacement in 2020 and 2021). The top 64 were chosen because at the end of each year, they receive a PDC-tour card and are allowed to compete in PDC Pro Tour events. The top 32 were chosen because these players automatically qualify for some major tournaments.

A third analysis only included players that have competed in all three audience conditions throughout 2018–2021, to test the robustness of the estimates of the audience conditions and to eliminate the variability of players without datapoints for a simulated audience and/or a real audience. Each player in the sample has competed in each respective audience condition at least once.

To further analyze the effects of a real audience a fourth analysis has been carried out, which only includes players who have competed in both audience conditions (no audience and real audience). This analysis has been carried out to get estimates on performances unrelated to the performances with a simulated audience.

The eliminate a bias throughout the years, a fifth analysis has been conducted including players in the top 64 in the Order of Merit who have competed in all three audience conditions for the year 2020, as 2020 included all three audience conditions unlike the other years, which only included no audience and either real or simulated audience.

### Sample

The data consisted of *N* = 26,724 individual performances of *k* = 442 players, who competed in 136 PDC ranking tournaments from January 2018 to March 2021. Out of the *k* = 442 players, five (1.13%) were women. On average each player competed in *m* = 64.6 matches, with a median of *mdn* = 19 games. Players ranged from only having a single data point, i.e., the player only played a single game, to a maximum of 431 individual performances over multiple tournaments and years. Players were ranked in the so-called Order of Merit and the Pro Tour Order of Merit (Pro Tour ranking). A player’s rank ranged from no rank (first year of competing on a professional level) to 223 for the Pro Tour ranking and no rank to 219 for the Order of Merit. The average Pro Tour rank was *m* = 53 with a median of *mdn* = 41. The average Order of Merit rank was *m* = 56 with a median of *mdn* = 44.

Descriptive statistics for data availability and the performance parameters 3DA and CP over the 4 years can be found in Table 1. Performance predictors 3DA and CP correlated as expected with *r* = .41 over the 4 years. The Order of Merit ranking and the Pro Tour ranking correlated with *r* = .90.

**Table 1:** Descriptive statistics of data availability (cases and percentage), performance parameters (means and standard deviations) and player ranking (mean and median).

	2018	2019	2020	2021	Combined
Cases (*N*)	7564	9654	7058	2448	26,724
*n*_no audience_	5584 (73.82%)	7618 (78.91%)	5806 (82.26%)	2024 (82.68%)	21,032 (78.70%)
*n*_simulated audience_	0 (0%)	0 (0%)	374 (5.30%)	424 (17.32%)	798 (2.99%)
*n*_real audience_	1980 (26.18%)	2036 (21.09%)	878 (12.44%)	0 (0%)	4894 (18.31%)
Average					
No audience	91.14 (6.89)	91.76 (6.87)	92.15 (6.90)	92.87 (6.58)	91.80 (6.87)
Simulated audience	*na*	*na*	95.39 (6.02)	93.20 (6.17)	94.21 (6.19)
Real audience	92.46 (6.92)	92.63 (6.83)	92.60 (6.40)	*na*	92.56 (6.79)
Checkout percentage					
No audience	40.75 (18.00)	41.62 (18.41)	41.43 (18.14)	42.12 (18.15)	41.39 (18.21)
Simulated audience	*na*	*na*	41.23 (16.88)	39.45 (13.74)	40.28 (15.31)
Real audience	39.28 (16.50)	39.25 (16.09)	38.93 (15.00)	*na*	39.19 (16.05)
Pro tour rank					
No audience	59 (46)	61 (48)	52 (42)	49 (42)	57 (45)
Simulated audience	*na*	*na*	31 (20)	44 (27)	23 (27)
Real audience	36 (24)	38 (25)	45 (32)	*na*	39 (26)
Order of merit rank					
No audience	64 (50)	64 (50)	56 (46)	56 (44)	61(48)
Simulated audience	*na*	*na*	31 (19)	49 (38)	40 (29)
Real audience	41 (25)	43 (29)	47 (33)	*na*	43 (29)

## Results

### Modelling results

#### Checkout percentage

The “random intercept model” for checkout performance indicated that clustering was present at the player level, justifying the use of multilevel modeling, *var*($\widehat{u}$_*1j*_) = 7.94, χ^2^ = 120.59, *p* < .001, ICC = .03. The second level (year) did not account for any clustering, *var*($\widehat{u}$_*2j*_) = 0.30, χ^2^ = 0.388, *p* > .05, ICC = 0, and was omitted from further analyses for simplicity.

Results from the final model indicate that playing with a simulated audience resulted in a decrease in CP, *b*_*sim*_ = − 2.48, *t*(22,838) = − 3.65*, p* < .001, with a partially standardized β_*stand_sim*_ = − .14, compared to no audience (see Table 2). A real audience resulted in larger performance decrements, *b*_*real*_ = − 3.62, *t*(22,835) = − 11.14*, p* < .001, β_*stand_real*_ = −  .20. The partially standardized beta coefficients can be considered small in effect size^60^.

**Table 2:** HLMs for CP, 3DA without CP as predictor, and 3DA with CP as predictor variable. β is partially standardized (*k* = 240).

Variables	Full model: CP			Full model: 3DA			HLM for 3DA (including CP as a control variable)		
Fixed effects	*b*	*SE*	β	*b*	*SE*	β	*b*	*SE*	β
Intercept	43.67***	(0.41)		93.82***	(0.37)		88.17***	(0.30)	
Audience (Ref: no audience)									
Real audience	− 3.62***	(0.33)	− .20	− 0.97***	(0.11)	− .14	− 0.49***	(0.10)	− .07
Simulated audience	− 2.48***	(0.68)	− .14	0.18	(0.23)	.03	0.54*	(0.21)	.08
Round	− 0.01***	(0.003)	− .0007	− 0.01***	(0.001)	− .002	− 0.01***	(0.0009)	− .002
Pro-tour rank	− 0.02***	(0.004)	− .0014	− 0.03***	(0.003)	− .005	− 0.03***	(0.003)	− .005
Checkout percentage							0.13***	(0.002)	.02

Random effects	Variance components	SD	ICC	Variance components	SD	ICC	Variance components	SD	ICC
Player	4.73	2.18	.01	0.12	0.33	.08	0.06	0.24	.08
Year				3.27	1.80	.12	0.09	0.29	.12
Residuals	301.21	17.36		0.69	0.83		0.58	0.76	
LogLikelihood		− 97,675.2			− 72,719.3			− 26,709.5	
Observations (*n*)		22,839			22,839			22,839	

Significance codes: *p < .05, **p < .01, ***p < .001.

The control variables “round of the tournament”, *b*_*round*_ = − 0.013, *t*(22,325) = − 4.53, *p* < .001, , and the level-two predictor “Pro Tour Order of Merit”, *b*_*rank*_ = − 0.024, *t*(390) = − 6.38*, p* < .001, significantly impacted CP. Since both ranking systems correlated with *r* = .90, *p* < .001 and to avoid multicollinearity, Pro Tour Order of Merit was chosen as an operationalization of player ranking, as it improved model fit more than the Order of Merit. The partially standardized coefficients indicated negligible effect size for round, β_*stand_round*_ = − .0007, and for rank, β_*stand_rank*_ = − .0014. With ICC = .016, less than 2% of the variance can be attributed to the variance between the players. The total variance explained according to Snijders and Bosker^61^ is *R*^2^ (S&B) = 3%. An effect size relating to the variance explained of the model, indicated a small effect according to Cohen^62^, *f*^*2*^ = 0.03.

#### Three-dart average

The “random intercept model” for 3DA indicated that significant clustering was present in the data. The residual variance at the individual level was *var*($\widehat{u}$_*0j*_) = 34.09, *p* < .05. Level two (year), *var*($\widehat{u}$_*1j*_) = 2.57, χ^2^ = 568.32, *p* < .001, and level three (players) *var*($\widehat{u}$_*2j*_) = 18.58, χ^2^ = 448.52, *p* <  .001, were significant. The final model with all audience conditions and predictor variables is summarized in Table 2. Playing with a simulated audience resulted in an increase in three-dart average, *b*_*sim*_ = 0.54, *t*(22,139) = 2.54*, p* < .01, whereas performing in front of a real audience players resulted in a performance decrease, *b*_*real*_ = − 0.49, *t*(22,726) = − 4.84*, p* < .001. Standardized coefficients of β_*stand_sim*_ =  .08 for simulated audiences and β_*stand_real*_ = − .07 for a real audiences can be considered negligible^60^.

Pro Tour Order of Merit, CP, and the round of the tournament were significant predictors of 3DA (all *p*s < .001, for effect size, see Table 2) and were added to the final model. The ICC = .07 at the second level indicated that roughly 7% of the total variance of 3DA can be explained by the year of performance. Thus, performances within each year were similar. Roughly 12% can be explained by the differences between the players (ICC = .12).

The total explained variance according to Snijders and Bosker^61^ is *R*^2^(S&B) = 42%, with a large effect size for variance explained in the overall model, *f*^*2*^ = 0.72^62^.

### Supplementary analyses

#### Top 64 & top 32

Subsamples of the top 32 of the Pro Tour Order of Merit and the top 64 of the Order of Merit have been analyzed (see Online Appendix 1–4 for descriptive statistics and model results). Both supplementary analyses display similar results as the main model, indicating that CP decreases with a simulated audience, top 32 CP β_*stand_sim*_ = − .17, top 64 CP β_*stand_sim*_ = − .17, and decreases further with a real audience, top 32 CP β_*stand_real*_ = − .22, top 64 CP β_*stand_real*_ = − .20. 3DA also decreased with a real audience, top 32 3DA β_*stand_real*_ = − .05, top 64 3DA β_*stand_real*_ = − .06, but increased with a simulated audience, top 32 3DA β_*stand_sim*_ = .08, top 64 3DA β_*stand_sim*_ = .12. These results and effect sized are similar to the main model.

#### All three audience conditions

Results from an analysis which only includes players who have competed with all three audience conditions, also indicate that CP decreases with a simulated audience, CP β_*stand_sim*_ = − .15, and further with a real audience, CP β_*stand_real*_ = − .20. 3DA decreased with a real audience, 3DA β_*stand_real*_ = − .07, and increased with a simulated audience, β_*stand_siml*_ = − .07. Again, these effect sizes closely resemble the main model (see Online Appendix 5 for the full model).

#### Effect of real and no audiences

Online Appendix 6 contains a model which excluded performances with simulated audiences to probe the effects of real audiences versus no audiences. Both performance indicators decrease with a real audience, CP β_*stand_real*_ = − .21, 3DA β_*stand_real*_ = − .05. Similar to the main model, this indicates the disruptive potential of real crowds on darts performance.

#### Top 64 in 2020

A sample that utilized strict criteria only including players in the top 64 who have competed under all three conditions in the year 2020, as in 2020 all three audience conditions were present, display a similar trend of the results for CP with decreases with simulated audience, CP β_*stand_sim*_ = − .15, and further decrease with a real audience, CP β_*stand_real*_ = − .19. The audience effects for average did not reach statistical significance, in this smaller sample, 3DA β_*stand_sim*_ = − .06, *p* = .21, 3DA β_*stand_real*_ = − .03, *p* = .42 (see Online Appendix 7 for the full model).

## Discussion

The goal of the current investigation was to answer the question how a real and a simulated audience influence the performance in the accuracy-based coordination-task darts. Competitions played without an audience were compared to competitions played with a real and a simulated audience.

The results indicate that players perform worse in the presence of a real audience than when performing alone. CP was highest when no audience was present, significantly decreased with artificial crowd noise (∆CP = 2.5%) and decreased further in the presence of a real audience (∆CP = 3.6%). However, effect sizes were small and the ICC of 1.6% indicated that within-player variability is high. The predictors all reached statistical significance, probably because of the large *N*, but with limited practical significance, i.e., small effect sizes (β_*stand_sim*_ = − .14, β_*stand_real*_ = − .20).

The results pattern of the 3DA variable are slightly different: while players perform worse with a real audience, they appear to perform better when playing with a simulated audience (baseline: no audience). The absolute difference in 3DA, however, is negligibly small, with only half a point decrease with a real audience and increase with a simulated audience, which is practically irrelevant for winning in darts. Small effect sizes are in line with many studies on audience effects in other sports^5,32^. The hierarchical linear model for 3DA could explain more than 40% of the total variance in 3DA with an ICC of 12%. Therefore, skill differences between the players can be assessed more accurately using 3DA as a performance predictor compared to CP.

Studies on social influences of spectators on performances in motor tasks could often identify small differences and effect sizes only^30^. However, it is worth explaining the present result pattern based on the most prominent theories.

In line with some social-facilitation theories and their predictions in motor tasks^31,32^ players were performing highest when playing without a real audience (however, to a small degree only). Findings for both performance predictors (CP and 3DA) were also evident in smaller samples of higher skill (i.e., players in the top 64 in the Order of Merit and top 32 of the Pro Tour Order of Merit), thus indicating that the audience effect is evident even in higher skilled players (see Online Appendix 3, 4), or in samples that applied more strict inclusion criteria (see Online Appendix 5–7).

These results help understand the effects of audiences in individual coordination-based sports: the presence of an audience decreases performance in a coordination-based sports task with high accuracy requirements. Several theories on the detrimental effects of an audience have been proposed in previous studies^5^. The deviation from the standard level of spectators (which for darts would be the no-audience condition, as most games are played behind closed doors) could lead to higher levels of social pressure and increase the likelihood of choking.

Therefore, the present results could be interpreted as a decrease in performance due to social pressure, rather than an increase in performance due to social support. Research suggests that choking can occur when performing in front of a crowd^6,63^, which has been shown in darts^46^. However, other studies in darts could not show relevant decrements, especially not in samples made up from professional players^23,25^.

Other theories emphasize the role of attentional resources: darts performance might decrease in the presence of spectators because the performer shifts their attention to the external distractor^35,37^. The audience acts as a distraction to the player, which taxes attentional resources, which are then no longer available for performing the main task, as Manstead and Semin^37^ suggested in the capacity hypothesis.

Alternatively, the audience could lead the performer to focus internally on movement execution, which disrupts the automated processes acquired through practice. This process is called “reinvestment”, which states that automated movement executions suffers when performers actively monitor the previously automated movement^64^. Reinvestment has often been linked to performance decrements under pressure, as it results in a disruption and in turn impairs performance, in this case, with a real audience present^65^. The simulated-audience situation might be perceived as less threatening, yet there still exists some distractive potential in comparison to when performing alone, especially if accidents with the audio occur^48^.

Otherwise, skill requirements for different aspects in the game of darts could be an explanation for the present result pattern. If CP decreases in conditions with a real and simulated audience, the scoring average (darts that are not aimed at a double, i.e., darts that are used to score as many points as possible) needs to be higher in conditions with a simulated audience to equalize the drop in CP. Scoring is a highly routinized task that players practice several hours per day. Elite players have mastered timing and minimalized errors^3^. Therefore, the movement should be highly refined and more sustainable under pressure than throwing at a double, as only a small portion of darts during a game are aimed at doubles, and the doubles that are played in a match do not necessarily have to be the same each leg that is played. Research by Ötting et al.^66^ on hot streaks or hot hands in darts has indicated that when players throw three darts in succession, they tend to have similar performances, which could influence the scoring, since the player can follow a good first dart with similar throws when aiming for a treble field. This would be impossible when throwing for a double, because as soon as the double is hit, the leg ends.

### Implications

Future research could benefit from analyzing the scoring average, which is the 3DA played without the throws that are aimed at a double field, thus independent from CP, or analyze newer, more predictive performance indicators, such as ordinal checkout efficiency, which is 40% more accurate in predicting wins than the 3DA^67^. Newer parameters with more predictive power should allow for a deeper understanding of (darts) performance under pressure and in turn increase our understanding of how performance changes in front of an active crowd and in pressure situations.

Future research in darts should also investigate whether specific crowd behaviors (e.g., supportive, hostile, neutral) leads to consecutive changes in performances. This could be interesting both on the level of overall play, or on the level of specific parts of the game. Outside of darts, there are few experimental^40^ or longitudinal within-game analyses (e.g., American football^68^; Basketball^69,70^) that generally show that motor performances do not relate to the behavior of the crowd. Archival studies are not the most controlled way to investigate the impact of crowd behavior, because such information is usually not included in the recorded data (see Humphreys et al.^71^ for a recent exception and approach), and the behavior of the crowd has to be monitored continuously (with small time intervals as e.g., described in van Meurs et al.^72^).

### Limitations

There are limitations to the current research that need to be noted and discussed. Several predictor variables were added to model the outcome on performance variables, yet there are more potential variables that need to be considered. The ranking points or prize money for each individual game varies, thus creating differently high incentives to perform well. Adding the guaranteed prize money earned for each respective game would allow to make inferences about the impact of financial incentives on darts performance. Apart from personal variables, such as personal stress, personal expectations and mental health, which are very hard to control for, the performance of the opponent has also not been considered. Players could be playing better or worse depending on the performance of the opponent. This “automated matching”^73^ could occur so that players would have equally high or low averages or checkout performances with regard to the opponent’s performance. Another variable that could increase the predictive power of the model is the duration of each match. Playing a match to six legs might differ to playing a match to 15 legs, as the players have more throws, thus regressing to their mean performance. This should be considered in future research, as including these variables could add to the predictive power of the model.

The inclusion of all players who have competed in PDC ranking tournaments can be seen as a limitation as well. Including individuals with only a few tournament matches could influence the effect size estimates, due to outliers, which would cause the model results to be less robust. We decided to include these players in the main analysis as we were interested in displaying the entire population of players who competed in ranking tournaments, and multilevel modelling was used to control for nested performances in individuals, which can utilize data from incomplete observations^74^. Supplementary analyses with stricter exclusion criteria in the Online Appendix indicate that results for subsamples are similar, however, future (experimental) research may benefit from more equal distributions across conditions, and from including a reliability factor of each player’s performance average.

While 3DA is currently the best indicator of darts performance, it does have limitations (i.e., the dependance on the performance of the opponent or CP). Therefore, the study would have benefitted from using more predictive measures of darts performance. However, these measures are still under development and currently not accessible.

### Conclusion

The current research offers insight into the effects of the presence of different types of audiences on performance in the coordination-based accuracy task darts. The results indicate that darts performance decreases in the presence of a real audience. Following this study result and returning to the introduction, the attempt to block the crowd distraction by using hearing protection could be beneficial, however, it would only be successful if wearing the protection would not distract the player itself, which would require specific training with protection on its own. Potentially, Gerwyn Price was not only distracted by the crowd, but also by his not well-fitting hearing protection. The case sparked interest in the media and as a reaction to the incident, the PDC has implemented a change of the rules, which now forbids players from wearing on-ear hearing protection^75^.

The results of this study indicate that a simulated audience has an equivocal effect on darts performance, impacting distinct areas within the game differently. Analyzing the impact of a simulated audience in other sports would further enlighten the effect of simulated crowds on performance in various sports tasks.

The performance differences among the three audience conditions are small and should not be overstated. Regardless of the approach taken, i.e., looking at the small effect sizes, versus looking at the significant differences in the audience conditions, playing professional darts in front of a real crowd does not lead to better performances, and a simulated crowd does not lead to significantly worse performances.

## Supplementary Information

Supplementary Information.

## Data Availability

The data that support the findings of this study are available from dartsorakel.com, where they can be accessed openly. For further questions regarding data and data availability please contact the corresponding author of this paper: Jona Greve, jona.greve@uni-muenster.de.

## References

[1] James, B. 2022. Why is Gerwyn Price being booed at the PDC darts?—Wales Online. https://www.walesonline.co.uk/sport/gerwyn-price-being-booed-pdc-22618004.

[2] Marland, D. 2023. Gerwyn Price Wears Headphones During PDC World Darts Championship Quarter-Final. https://www.sportbible.com/other/breaking-gerwyn-price-headphones-pdc-world-darts-championship-476082-20230101.

[3] Smeets JBJ, Frens MA, Brenner E (2002). Throwing darts: Timing is not the limiting factor. Exp. Brain Res..

[4] Phillips, J. 2020. Wright’s Reign Ended by Clemens as World Championship resumes | PDC. *pdc.tv* https://www.pdc.tv/news/2020/12/27/wrights-reign-ended-clemens-world-championship-resumes.

[5] Strauss, B., Staufenbiel, K., van Meurs, E. & MacMahon, C. Social influence of sport spectators. In *Sport and Exercise Psychology* (eds Schüler, J., Wegner, M., Plessner, H. & Eklund, R. C.) (Springer, 2023).

[6] Wallace HM, Baumeister RF, Vohs KD (2005). Audience support and choking under pressure: A home disadvantage?. J. Sports Sci..

[7] TOKYO2020. 2020. Olympic Games Postponed to 2021. *TOKYO2020. News. General* https://olympics.com/tokyo-2020/en/news/joint-statement-from-international-olympic-committee-and-tokyo2020.

[8] Lam, N. 2021. Tokyo 2020 Olympics: Will Empty Stadiums and Lack of Fans Affect Athletes? | South China Morning Post. 2021 https://www.scmp.com/week-asia/health-environment/article/3141462/tokyo-2020-olympics-will-empty-stadiums-and-lack-fans.

[9] Leota J (2021). Home is where the hustle is: The influence of crowds on effort and home advantage in the National Basketball Association. SSRN Electron. J..

[10] Bryson A, Dolton P, Reade JJ, Schreyer D, Singleton C (2021). Causal effects of an absent crowd on performances and refereeing decisions during Covid-19. Econ. Lett..

[11] Leitner MC, Daumann F, Follert F, Richlan F (2022). The cauldron has cooled down: A systematic literature review on home advantage in football during the COVID-19 pandemic from a socio-economic and psychological perspective. Manag. Rev. Q..

[12] Benz LS, Lopez MJ (2021). Estimating the change in soccer’s home advantage during the Covid-19 pandemic using bivariate Poisson regression. AStA Adv. Stat. Anal..

[13] Bilalić M, Gula B, Vaci N (2021). Home advantage mediated (HAM) by referee bias and team performance during covid. Sci. Rep..

[14] Chiu YC, Chang CK (2022). Major League Baseball during the COVID-19 pandemic: Does a lack of spectators affect home advantage?. Humanit. Soc. Sci. Commun..

[15] Lu P (2022). Impact of COVID-19 lockdown on match performances in the National Basketball Association. Front. Psychol..

[16] Higgs N, Stavness I (2021). Bayesian analysis of home advantage in North American professional sports before and during COVID-19. Sci. Rep..

[17] Szabó DZ (2022). The impact of differing audience sizes on referees and team performance from a North American perspective. Psychol. Sport Exerc..

[18] Fazackerley LA, Gorman AD, Minett GM, Caia J, Kelly VG (2022). The influence of absent crowds on National Rugby League match player statistics and running metrics. Psychol. Sport Exerc..

[19] Ferraresi M, Gucciardi G (2021). Who chokes on a penalty kick? Social environment and individual performance during Covid-19 times. Econ. Lett..

[20] Fleishman EA (1957). Factor structure in relation to task difficulty in psychomotor performance. Educ. Psychol. Meas..

[21] Zeniya H, Tanaka H (2021). Effects of different types of analogy instruction on the performance and inter-joint coordination of novice darts learners. Psychol. Sport Exerc..

[22] Tran BN, Yano S, Kondo T (2019). Coordination of human movements resulting in motor strategies exploited by skilled players during a throwing task. PLoS ONE.

[23] Ötting M (2020). Performance under pressure in skill tasks: An analysis of professional darts. PLoS ONE.

[24] Mawer, N., Ollerenshaw, T., Titmus, M., Gardner, R. & Marsh, J. *Darts Regulation Authority Rulebook*. www.thedra.co.uk (2019).

[25] Klein Teeselink B, Potter van Loon RJD, van den Assem MJ, van Dolder D (2020). Incentives, performance and choking in darts. J. Econ. Behav. Organ..

[26] Liebscher S, Kirschstein T (2017). Predicting the outcome of professional darts tournaments. Int. J. Perform. Anal. Sport.

[27] Cottrell, N. B. Social Facilitation. in *Experimental Social Psychology* (ed. McClintock, C. G.) 185–236 (Holt, Rinehart and Winston, 1972).

[28] Zajonc RB (1965). Social facilitation. Science (80-).

[29] Landers D, McCullagh PD (1976). Social facilitation of motor performance. Exerc. Sport Sci. Rev..

[30] Bond CF, Titus LJ (1983). Social facilitation: A meta-analysis of 241 studies. Psychol. Bull..

[31] Strauss B (2002). Social facilitation in motor tasks: A review of research and theory. Psychol. Sport Exerc..

[32] van Meurs, E., Greve, J. & Strauss, B. Moving in the presence of others—A systematic review and meta-analysis on social facilitation. *Int. Rev. Sport Exerc. Psychol.* **1**, (2022).

[33] Henchy T, Glass DC (1968). Evaluation apprehension and the social facilitation of dominant and subordinate responses. J. Pers. Soc. Psychol..

[34] Guerin B, Innes JM (1982). Social facilitation and social monitoring: A new look at Zajonc’s mere presence hypothesis. Br. J. Soc. Psychol..

[35] Sanders GS, Baron RS, Moore DL (1978). Distraction and social comparison as mediators of social facilitation effects. J. Exp. Soc. Psychol..

[36] Baron RS (1986). Distraction-conflict theory: Progress and problems. Adv. Exp. Soc. Psychol..

[37] Manstead ASR, Semin GR (1980). Social facilitation effects: Mere enhancement of dominant responses?. Br. J. Soc. Clin. Psychol..

[38] Edwards AM, Dutton-Challis L, Cottrell D, Guy JH, Hettinga FJ (2018). Impact of active and passive social facilitation on self-paced endurance and sprint exercise: Encouragement augments performance and motivation to exercise. BMJ Open Sport Exerc. Med..

[39] Harb-Wu K, Krumer A (2019). Choking under pressure in front of a supportive audience: Evidence from professional biathlon. J. Econ. Behav. Organ..

[40] Epting LK, Riggs KN, Knowles JD, Hanky JJ (2011). Cheers versus jeers: Effects of audience feedback on individual athletic performance. N. Am. J. Psychol..

[41] Baumeister RF (1984). Choking under pressure: Self-consciousness and paradoxical effects of incentives on skillful performance. J. Pers. Soc. Psychol..

[42] Beilock SL, Afremow JA, Rabe AL, Carr TH (2001). ‘Don’t miss!’ the debilitating effects of suppressive imagery on golf putting performance. J. Sport Exerc. Psychol..

[43] Gröpel P, Mesagno C (2019). Choking interventions in sports: A systematic review. Int. Rev. Sport Exerc. Psychol..

[44] Rupprecht, A. G. O., Tran, U. S. & Gröpel, P. The effectiveness of pre-performance routines in sports: a meta-analysis. *Int. Rev. Sport Exerc. Psychol.* **0**, 1–26 (2021).

[45] Low WR (2021). Pressure training for performance domains: A meta-analysis. Sport. Exerc. Perform. Psychol..

[46] Goller D (2022). Analysing a built-in advantage in asymmetric darts contests using causal machine learning. Ann. Oper. Res..

[47] Lanning, P. 2020. Darts World—Gurney hits out at fake crowd noise. https://dartsworld.com/content/article/gurney-hits-out-at-fake-crowd-noise.

[48] Gill, S. 2020. Sky Sports offers apology to Lauby after bad timed mistake with crowd noise | Dartsnews.com. https://dartsnews.com/pdc/sky-sports-offers-apology-to-lauby-after-bad-timed-mistake-with-crowd-noise.

[49] Hillier, B. 2020. Can artificial crowd noise match the thrill of packed stadiums? | The World from PRX. The World https://theworld.org/stories/2020-08-04/can-artificial-crowd-noise-match-thrill-packed-stadiums.

[50] Marland, D. 2021. Sky Sports ‘Mute’ Crowd After X-Rated Gerwyn Price Insult Leaves Viewers Stunned. https://www.sportbible.com/other/sky-sports-mute-darts-crowd-after-xrated-gerwyn-price-insult-20211229.

[51] Wickham, H. & Wickham, M. H. 2016. *Package ‘rvest’*.

[52] Darts Orakel. 2021. Nine-darter Library, Super Stats and Jose FDI #1. https://mailchi.mp/eb04ea6d9274/nine-darter-library-super-stats-and-jose-fdi-1?e=e8a52c6872.

[53] darts1.de. 2021. PDC Pro Tour Order of Merit. https://www.darts1.de/ranglisten/PDC-Pro-Tour-Order-of-Merit.php.

[54] Nicholson, P. 2021. Is the PDC Order of Merit a ‘fair’ World Ranking System for Darts or is it Time for Change? https://www.sportinglife.com/darts/news/time-for-ranking-system-to-change/190881?aff=1197317995&dcmp=SL_FACEBOOK_RANKINGS.

[55] Phillips, J. 2018. World Record Crowd At German Darts Masters | PDC. https://www.pdc.tv/news/2018/06/14/world-record-crowd-german-darts-masters.

[56] Field A (2009). Discovering Statistics using SPSS.

[57] Hox JJ, Moerbeek M, de Schoot R, Maas CJM (2017). Multilevel Analysis: Techniques and Applications.

[58] Bates, D., Mächler, M., Bolker, B. M. & Walker, S. C. Fitting linear mixed-effects models using lme4. *J. Stat. Softw.* **67**, (2015).

[59] Lorah J (2018). Effect size measures for multilevel models: Definition, interpretation, and TIMSS example. Large-Scale Assess. Educ..

[60] Fey CF, Hu T, Delios A (2022). The measurement and communication of effect sizes in management research. Manag. Organ. Rev..

[61] Snijders TAB, Bosker RJ (1994). Modeled variance in two-level models. Soc. Methods Res..

[62] Cohen J (1992). A power primer. Psychol. Bull..

[63] Jane WJ (2022). Choking or excelling under pressure: Evidence of the causal effect of audience size on performance. Bull. Econ. Res..

[64] Jackson RC, Ashford KJ, Norsworthy G (2006). Attentional focus, dispositional reinvestment, and skilled motor performance under pressure. J. Sport Exerc. Psychol..

[65] Beilock SL, Jellison WA, Rydell RJ, McConnell AR, Carr TH (2006). On the causal mechanisms of stereotype threat: Can skills that don’t rely heavily on working memory still be threatened?. Personal. Soc. Psychol. Bull..

[66] Ötting, M., Langrock, R., Deutscher, C. & Leos-Barajas, V. *The hot hand in professional darts*. *arXiv* vol. 183 565–580 (Blackwell Publishing Ltd, 2018).

[67] Darts Orakel. 2021. Which Belgian Gave a Finishing Masterclass? OChE Rating Explained. https://mailchi.mp/5716a16b4583/which-belgian-gave-a-finishing-masterclass-oche-rating-explained?e=e8a52c6872.

[68] Strauss B (2002). The impact of supportive spectator behavior on performance in team sports. Int. J. Sport Psychol..

[69] Thirer J, Rampey MS (1979). Effects of abusive spectators’ behavior on performance of home and visiting intercollegiate basketball teams. Percept. Mot. Skills.

[70] Greer DL (1983). Spectator booing and the home advantage: A study of social influence in the basketball arena. Soc. Psychol. Q..

[71] Humphreys BR, Reade J, Schreyer D, Singleton C (2022). Separating the crowds: Examining home and away attendances at football matches. SSRN Electron. J..

[72] van Meurs E, Vergeld V, Strauss B, Gehrau V (2022). Die Beobachtung als Methode in der Sportwissenschaft. Die Beobachtung als Methode in der Sportwissenschaft.

[73] Forgas JP, Brennan G, Howe S, Kane JF, Sweet S (1980). Audience effects on squash players’ performance. J. Soc. Psychol..

[74] Finch, W. H., Bolin, J. E. & Kelley, K. Multilevel modeling using R. *Multilevel Model. Using R* 1–207 (2016) 10.1080/00949655.2020.1805564.

[75] Walters, M. 2023. Gerwyn Price Banned from Repeating Headphones Stunt as Premier League Changes Rules—Daily Star. https://www.dailystar.co.uk/sport/darts/gerwyn-price-headphones-banned-premierleague-29081144.

