# Supplementary material for: Elite darts performance and the social influence of real crowds and simulated crowd noise
Source: Sci Rep. 2023 Jul 31;13:12346. doi: 10.1038/s41598-023-39378-x (PMC10390587; doi:10.1038/s41598-023-39378-x)
Supplement: Supplementary file 1 — Supplementary Information. [file 41598_2023_39378_MOESM1_ESM.docx]

***Appendix***

Appendix 1 – Descriptive statistics for players with an Order of Merit rank of 64 or higher (cases and percentages), performance parameters (means and standard deviations) and player ranking (mean and median).

|  | **2018** | **2019** | **2020** | **2021** | **Combined** |
| --- | --- | --- | --- | --- | --- |
| **Cases (*N*)** | 4,394 | 5,205 | 4,018 | 1,345 | 14,906 |
| *n*_no audience_ | 2,990 (68.05%) | 3,821 (73.41%) | 3,148 (78.35%) | 1,093 (81.26%) | 11,052 (73.87%) |
| *n*_simulated audience_ | 0 (0%) | 0 (0%) | 307 (7.64%) | 252 (18.74%) | 559 (3.74%) |
| *n*_real audience_ | 1,404 (31.95%) | 1,384 (26.59%) | 563 (14.01%) | 0 (0%) | 3351 (22.39%) |
| **Average** |  |  |  |  |  |
| No audience | 92.87 (6.65) | 93.73 (6.46) | 94.24 (6.50) | 94.84 (6.27) | 93.75 (6.53) |
| Simulated audience | *na* | *na* | 96.08 (5.65) | 95.75 (5.20) | 95.93 (5.45) |
| Real audience | 94.00 (6.42) | 94.12 (5.95) | 94.53 (5.88) | *na* | 94.14 (6.14) |
| **Checkout Percentage** |  |  |  |  |  |
| No audience | 42.22 (17.35) | 42.91 (17.36) | 43.18 (17.45) | 43.24 (17.07) | 42.83 (17.36) |
| Simulated audience | *na* | *na* | 41.10 (15.81) | 40.69 (11.57) | 40.92 (14.10) |
| Real audience | 40.23 (16.10) | 40.21 (15.24) | 39.85 (13.98) | *na* | 40.16 (15.40) |
| **Pro Tour Rank** |  |  |  |  |  |
| No audience | 33 (26) | 32 (26) | 30 (24) | 29 (23) | 31 (25) |
| Simulated audience | *na* | *na* | 24 (18) | 28 (24) | 26 (20) |
| Real audience | 25 (20) | 22 (17) | 26 (20) | *na* | 24 (18) |
| **Order of Merit Rank** |  |  |  |  |  |
| No audience | 30 (29) | 29 (29) | 28 (26) | 28 (27) | 29 (28) |
| Simulated audience | *na* | *na* | 21 (15) | 26 (26) | 24 (19) |
| Real audience | 24 (20) | 22 (16) | 25 (20) | *na* | 23 (19) |

Appendix 2 – Descriptive statistics for players with an Order of Merit rank of 32 or higher (cases and percentages), performance parameters (means and standard deviations) and player ranking (mean and median).

|  | **2018** | **2019** | **2020** | **2021** | **Combined** |
| --- | --- | --- | --- | --- | --- |
| **Cases (*N*)** | 2,779 | 3,414 | 2,652 | 867 | 9,712 |
| *n*_no audience_ | 1,765 (63.51%) | 2,338 (68.48%) | 2,028 (76.47%) | 707 (81.55%) | 6835 (70.41%) |
| *n*_simulated audience_ | 0 (0%) | 0 (0%) | 244 (9.20%) | 160 (18.45%) | 404 (4.16%) |
| *n*_real audience_ | 1,014 (36.49%) | 1,076 (31.52%) | 380 (14.33%) | 0 (0%) | 2470 (25.43%) |
| **Average** |  |  |  |  |  |
| No audience | 93.86 (6.70) | 95.10 (6.23) | 95.34 (6.25) | 95.98 (6.14) | 94.94 (6.39) |
| Simulated audience | *na* | *na* | 96.30 (5.79) | 96.28 (5.36) | 96.29 (5.62) |
| Real audience | 94.86 (6.33) | 94.91 (5.77) | 95.57 (5.46) | *na* | 95.00 (5.96) |
| **Checkout Percentage** |  |  |  |  |  |
| No audience | 42.63 (17.10) | 43.90 (16.87) | 44.21 (16.74) | 44.62 (16.37) | 43.74 (16.84) |
| Simulated audience | *na* | *na* | 41.76 (16.08) | 41.50 (11.23) | 41.66 (14.34) |
| Real audience | 40.96 (15.89) | 40.66 (15.42) | 41.17 (14.21) | *na* | 40.86 (15.43) |
| **Pro Tour Rank** |  |  |  |  |  |
| No audience | 16 (16) | 16 (17) | 15 (14) | 15 (16) | 16 (16) |
| Simulated audience | *na* | *na* | 15 (13) | 15 (16) | 15 (14) |
| Real audience | 13 (12) | 13 (11) | 13 (12) | *na* | 13 (11) |
| **Order of Merit Rank** |  |  |  |  |  |
| No audience | 18 (18) | 21 (16) | 19 (16) | 22 (17) | 20 (17) |
| Simulated audience | *na* | *na* | 17 (14) | 21 (17) | 18 (15) |
| Real audience | 16 (16) | 17 (12) | 16 (12) | *na* | 16 (13) |

Appendix 3 – Multilevel models for CP and 3DA for the top 64 in the Order of Merit ranking. β is partially standardized (*k*=86).

| **Variables** | **TOP 64 CP** | | | **TOP 64 3DA** | |  |
| --- | --- | --- | --- | --- | --- | --- |
| **Fixed effects** | *b* | *SE* | β | *b* | *SE* | β |
| Intercept | 44.54*** | (0.50) |  | 88.64*** | (0.34) |  |
| Audience (*Ref:* no audience) |  |  |  |  |  |  |
| Real audience | -3.85*** | (0.36) | -.20 | -0.46*** | (0.12) | -.06 |
| Simulated audience | -3.10*** | (0.74) | -.17 | 0.70** | (0.24) | .12 |
| Round | -0.01*** | (0.003) | -.0006 | -0.01*** | (0.001) | -.002 |
| Pro tour rank | -0.03*** | (0.001) | -.0027 | -0.03*** | (0.005) | -.007 |
| Checkout percentage |  |  |  | 0.15*** | (0.003) | .02 |
| **Random effects** | *Variance*  *components* | *SD* | *ICC* | *Variance*  *components* | *SD* | *ICC* |
| Player | 3.50 | 1.88 | .01 | 3.61 | 1.09 | .11 |
| Year |  |  |  | 1.83 | 1.36 | .05 |
| Residuals | 277.02 | 16.64 |  | 26.52 | 5.15 |  |
| Log-Likelihood |  | -63,112.1 |  |  | -45,835 |  |
| Observations (*n*) |  | 14,906 |  |  | 14,906 |  |
| Significance codes: **p* < .05, ***p* < .01, ****p* < .001 | | |  |  |  |  |

Appendix 4 – Multilevel models for CP and 3DA for the Top 32 in the Pro Tour Ranking. β is partially standardized (*k*=58).

| **Variables** | **TOP 32 CP** | | | **TOP 32 3DA** | |  |
| --- | --- | --- | --- | --- | --- | --- |
| **Fixed effects** | *b* | *SE* | β | *b* | *SE* | β |
| Intercept | 45.63*** | (0.61) |  | 89.34** | (0.51) |  |
| Audience (*Ref:* no audience) |  |  |  |  |  |  |
| Real audience | -3.83*** | (0.42) | -.22 | -0.33* | (0.14) | -.05 |
| Simulated audience | -2.96*** | (0.85) | -.17 | 0.56* | (0.28) | .08 |
| Round | -0.01*** | (0.003) | -.0008 | -0.01*** | (0.001) | -.002 |
| Pro tour rank | -0.08*** | (0.03) | -.0045 | -0.05** | (0.02) | -.008 |
| Checkout percentage |  |  |  | 0.15*** | (0.003) | .02 |
| **Random effects** | *Variance*  *components* | *SD* | *ICC* | *Variance*  *components* | *SD* | *ICC* |
| Player | 2.88 | 1.70 | .01 | 4.01 | 2.00 | .04 |
| Year |  |  |  | 1.22 | 1.10 | .13 |
| Residuals | 263.97 | 16.25 |  | 26.26 | 5.12 |  |
| LogLikelihood |  | -40,883.5 |  |  | -29,790.8 |  |
| Observations (*n*) |  | 9,712 |  |  | 9,712 |  |
| Significance codes: : **p* < .05, ***p* < .01, ****p* < .001 | | |  |  |  |  |

Appendix 5 – Multilevel models for CP and 3DA for players who have competed in all conditions at least once. β is partially standardized (*k*=113).

| **Variables** | **All 3 audience conditions CP** | | | **All 3 audience conditions 3DA** | |  |
| --- | --- | --- | --- | --- | --- | --- |
| **Fixed effects** | *b* | *SE* | β | *b* | *SE* | β |
| Intercept | 44.12*** | (0.42) |  | 88.90*** | (0.29) |  |
| Audience (*Ref:* no audience) |  |  |  |  |  |  |
| Real audience | -3.58*** | (0.34) | -.20 | -0.47*** | (0.11) | -.07 |
| Simulated audience | -2.60*** | (0.67) | -.15 | 0.51* | (0.21) | .07 |
| Round | -0.01*** | (0.003) | -.0005 | -0.01*** | (0.001) | -.001 |
| Pro tour rank | -0.03*** | (0.005) | -.001 | -0.03*** | (0.003) | -.004 |
| Checkout percentage |  |  |  | 0.14*** | (0.002) | .02 |
| **Random effects** | *Variance*  *components* | *SD* | *ICC* | *Variance*  *components* | *SD* | *ICC* |
| Player | 3.31 | 1.82 | .01 | 2.43 | 1.56 | .08 |
| Year |  |  |  | 1.78 | 1.34 | .06 |
| Residuals | 283.77 | 16.85 |  | 26.94 | 5.19 |  |
| LogLikelihood |  | -75,747.3 |  |  | -54,992.2 |  |
| Observations (*n*) |  | 17,840 |  |  | 17,840 |  |
| Significance codes: **p* < .05,***p* < .01, ****p* < .001 | | |  |  |  |  |

Appendix 6 – Multilevel models for CP and 3DA for players who have competed in with a real and no audience at least once. β is partially standardized (*k*=190).

| **Variables** | **Real and no audience CP** | | | **Real and no audience 3DA** | |  |
| --- | --- | --- | --- | --- | --- | --- |
| **Fixed effects** | *b* | *SE* | β | *b* | *SE* | β |
| Intercept | 45.80*** | (0.41) |  | 87.98*** | (0.30) |  |
| Audience (*Ref:* no audience) |  |  |  |  |  |  |
| Real audience | -3.65*** | (0.33) | -.21 | -0.45*** | (0.10) | -.05 |
| Round | -0.01*** | (0.03) | -.0008 | -0.01*** | (0.009) | -.002 |
| Pro tour rank | -0.02*** | (0.04) | -.0012 | -0.02** | (0.03) | -.008 |
| Checkout percentage |  |  |  | 0.14*** | (0.02) | .02 |
| **Random effects** | *Variance*  *components* | *SD* | *ICC* | *Variance*  *components* | *SD* | *ICC* |
| Player | 4.03 | 2.00 | .01 | 3.83 | 1.96 | .07 |
| Year |  |  |  | 2.41 | 1.55 | .11 |
| Residuals | 301.35 | 17.36 |  | 26.26 | 5.12 |  |
| LogLikelihood |  | -92,758.6 |  |  | -67,109.3 |  |
| Observations (*n*) |  | 21,690 |  |  | 21,690 |  |
| Significance codes: **p* < .05,***p* < .01, ****p* < .001 | | |  |  |  |  |

Appendix 7 – Multilevel models for CP and 3DA for top 64 players who have competed in all conditions in 2020 (*k*=53).

| **Variables** | **CP** | | | **3DA** | |  |
| --- | --- | --- | --- | --- | --- | --- |
| **Fixed effects** | *b* | *SE* | β | *b* | *SE* | β |
| Intercept | 45.03*** | (0.73) |  | 90.06*** | (0.29) |  |
| Audience (*Ref:* no audience) |  |  |  |  |  |  |
| Real audience | -3.43*** | (0.82) | -.19 | -0.21 | (0.26) | -.03 |
| Simulated audience | -2.70* | (1.04) | -.15 | 0.41 | (0.33) | .06 |
| Round | -0.001 | (0.01) | -.0006 | -0.01*** | (0.002) | -.002 |
| Pro tour rank | -0.07*** | (0.02) | -.004 | -0.03** | (0.01) | -.005 |
| Checkout percentage |  |  |  | 0.14*** | (0.01) | .02 |
| **Random effects** | *Variance*  *components* | *SD* | *ICC* | *Variance*  *components* | *SD* | *ICC* |
| Player | 2.77 | 1.66 | .01 | 3.27 | 1.81 | .11 |
| Residuals | 237.62 | 16.54 |  | 26.81 | 5.18 |  |
| LogLikelihood |  | -15,958.6 |  |  | -11,619.8 |  |
| Observations (*n*) |  | 3774 |  |  | 3774 |  |
| Significance codes: **p* < .05,***p* < .01, ****p* < .001 | | |  |  |  |  |
